# The Value of Drinking-Water as a Physiological and Therapeutic Agent*Read before the Augusta Academy of Medicine.

**Published:** 1897-12

**Authors:** A. J. Kilpatrick

**Affiliations:** Augusta, Ga.


### THE VALUE OF DRINKING-WATER AS A PHYSIOLOGICAL AND THERAPEUTIC AGENT.*

BY A. J. KILPATRICK, M.D.,

AUGUSTA, GA.

With the exception of air water is considered the most necessary adjunct of both animal and vegetable life. Possessing this wonderful field of usefulness, it is indeed strange that its physiological and therapeutical value should be so little studied and appreciated by medical men in endeavoring to find agents for the cure of diseased humanity.

Realizing as I do the neglect this subject is receiving, and believing as I do, not only in the usefulness but absolute necessity of this agent I present it as the subject of this evening's discussion. When I say "drinking-water," I mean neither chemically nor bacteriologically pure, but ordinary water as found in nature.

When we examine into human physiology water is found to be ever present in all fluids and tissues of the body, varying from

* Read before the Augusta Academy of Medicine.

2 per cent. in the enamel of the teeth to 99.5 in the saliva. The bones contain 13 per cent.; muscles 75 per cent.; blood 79.5 per cent.; bile 88 per cent.; gastric juice 98.6 per cent.; cartilage 55 per cent., etc. So, taking the body as a whole, we are composed of 60 parts water and 40 parts solids.

In studying the uses of water we find that it is, first, a solvent. It is this property above all others that gives it its true field of usefulness. Its solvent power, as we know, is almost universal. In entering the body with the food and acting together with the secretions, it brings the food into a suitable condition necessary to absorption. In this day of crackers, stale bread, and cold dry meals in general, it is a great tax we place upon the salivary glands in moistening this food sufficiently for its reception by the stomach; and also a tax upon the stomach and intestines to prepare it for absorption.

Being a solvent we cannot exactly separate the idea that water is, secondly, a common carrier, conveying food to and removing waste material from the tissues.

From these two properties we may conclude that water should be taken at and between meals in order that we may make the best use of our food. At meals, for the proper preparation and absorption; between meals, to aid the tissues in getting rid of their worn-out or offending material by the various channels of elimination.

In looking up the amount of water thrown off by these eliminating channels, we find 4 per cent. by the fauces, 20 per cent. by the lungs, 30 per cent. by the perspiratory glands, 46 by the kidneys. So we see by this solvent and transporting property water is nature's diuretic, diaphoretic and laxative.

Lastly, we find that water is a true constituent of tissue. It gives cartilage its elasticity, tendon its toughness and pliability, bone its peculiar power of resisting internal muscular strain and external violence. So, in the same way, it must be a constituent of blood and glandular secretions in order that their respective functions may be performed.

As the vegetable kingdom is dependent upon water for its proper growth and development, so is the animal. In fact, there is no vital action without water. Our bodies, in health, are, as we have seen, more fluid than solid, and if we are to keep in health this fluidity must be maintained. The amount necessary, of course, varies, depending upon bodily temperature, bodily work, etc., but five pints of water is considered by the physiologist to be the average quantity needed for an adult. One pint is said to be taken in the food, the remaining four pints, to be taken as drinking-water.

As we have studied something of the uses of water in health, let us make a few applications as to its uses in sickness. The idea so prevalent in past days that lying-in women and fever patients should not be allowed water still exists among the laity, and I am sorry to say to some extent in the medical profession. In my short expeence as a medical man how often have I heard this absurd idea advanced! It has been but a few weeks since I was called to see an old man, fast dying with an .ulcerated necrosed state of the alimentary tract, with temperature 104° Fah., accompanied by profuse perspiration. He was literally burning and sweating himself away. On finding these symptoms, and besides expressing himself as "burning up," I at once offered him a glass of cool water. On making him this offer, weak as he was, he assumed an expression of both surprise and gratitude; surprise, because I should offer him the thing above all others that his family and friends had impressed upon him was not only harmful but deadly; gratitude because he now knew that he could drink freely of that life-giving fluid for which every tissue of the body was crying, and to which every tissue of the body owed its vitality. How often is the question asked: "Can the patient have water?" And on receiving an affirmative answer will state that he or she has been begging for it, but "we were afraid to give it!" I have seen several women, most of them young women, complaining of dullness or fullness in the region of the stomach, headache, constipation and with a general dilapidated appearance, who, on being questioned as to drinking-water, will state that they never drink water because they have indigestion and water makes them worse; but that they drink instead coffee or tea. The trouble is this lack of water has produced the indigestion. They need water to increase pressure in the portal circulation, thereby washing the liver of its effete material and consequently increasing peristalsis, diuresis and diaphoresis, which have already been alluded to.

It may be deemed presumptuous for one just entering the medical field to express any positive view to this academy, but I believe that water is the thing above all others needed in all wasting diseases, as typhoid fever, tuberculosis and the like. While resident physician to the City Hospital of this city I had the good fortune to have control of three or four typhoid patients, whom I watched very closely. On noticing the constant sweat, frequently liquid and often bloody alvine discharges, dry tongue, parched lips, indicating that the body was being drained of its watery element, I came to the conclusion that water would be of more value than all our antipyretic, intestinal antiseptics, etc. In fact, I thought water would be an antipyretic, antiseptic, and at the same time supply the deficiency that was caused by this constant drainage. And used it accordingly with the best results. While extensive as I believe the field of application of this subject is, the literature bearing upon it is indeed limited, and with one exception, I have been totally unable to find anything that would aid me in expressing my views. That exception coming from the distinguished Dr. Meigs of Philadelphia, I am glad to quote him on this subject of typhoid fever. This eminent man insisted upon an abundant supply of water in typhoid fever. His experience, which was said to be unusually large, led him to believe that death was often due to want of water, and therefore constantly supplied it. I will read you a quotation from a lecture by Dr. Meigs, delivered at Pennsylvania Hospital in 1879:

"When I stand by the bedside of a typhoid fever patient, and see the patient motionless, insensible, dead to all the usual senses of the living; when I look at his half-closed eyes, his gaping mouth, his dried and furred tongue; when I brush the unheeded flies from his unconscious face, and when I touch his hot and burning skin, I ask myself into what lower state the human body can fall. Not only has the patient lost all appetite for food—not only is he dead to all that surrounds him, but this hot and withered body, this dry and pasty mouth, filled with desiccated crust and sordes, knows no longer even the sense of thirst. This has been the last sense of which he has been deprived. So long as he retained any consciousness at all he would ask for water or for ice. Now he feels not even this great want. It is at this crisis of his life that he is to be saved—if saved at all—only by the greatest care of his physician and nurses and relatives; and woe to the physician who can look on such a sight and not yearn to know all that his art has acquired through centuries of experience and study."

Dr. Meigs further says eight pints of water in twenty-four hours can be given with decided benefit. Now the question may arise in your minds whether this large assumption of water would not interfere with other and more nutritious substances being absorbed. Now, I think, the question of nourishment is a relative one entirely. To a body suffering for water nothing is so nutritiuos as water. We do not attribute the withering of vegetation, during the dry, "heated term" of summer so much to the heat as to the drought; and may we not have an analogous drought in our bodies during the "heated term" of fever?

For fear of consuming too much time, and knowing that the most instructive part of these meetings is that devoted to the discussion of the paper, I will not go into the many and varied conditions which I think would be benefited by free drinking of water. I will, however, mention that dreaded scourge of the human race, consumption.

The difference between a green leaf and a dry leaf, a living muscle and a desiccated muscle, needs no explanation. The cells of our body, as we have seen, are aquatic in their habit. The activity of all protoplasm depends upon a flux of its constituents; that flux must have a medium; that medium must be fluid, and that fluid must be water. Thus in all cases in which we desire to stimulate cell activity, as in consumption, we must supply water.

In the two diseases above mentioned, in conjunction with yellow fever, scarlet fever, etc., the system is supposed to be burdened with a poisonous product called toxin. May we not hope that water given freely has the power of washing this poisonous element from the system?

I know very little of the so-called medicinal springs of our country, and therefore cannot speak authoritatively upon the subject; but the benefit so often derived, in my opinion, is not from the medicinal ingredients within the water, but from the large amount of water taken.

I have not alluded to its refreshing quality. But is it not strange that we should slight the only invigorating, nourishing and life-giving beverage mother nature has given us?

Gentlemen, when we learn more of the physiological and therapeutical value of drinking-water we will have made a rapid stride in treating diseased humanity.

